# Lipid Peroxidation: Analysis and Applications in Biological Systems

**DOI:** 10.3390/antiox8020040

**Published:** 2019-02-13

**Authors:** Jetty Chung-Yung Lee, Thierry Durand

**Affiliations:** 1 School of Biological Sciences, The University of Hong Kong, Pokfulam Road, Hong Kong, China; 2 Institut des Biomolécules Max Mousseron (IBMM), UMR 5247, CNRS Université de Montpellier, ENSCM, F-34093 Montpellier, France; thierry.durand@umontpellier.fr

Lipid peroxidation is an ambiguous event in all biological species. Excessive accumulation in vivo is associated with disease development, whereas ex vivo build-up in food deteriorates the nutrient quality. To foot the balance and control the extent of lipid peroxidation, antioxidants are required to prevent some of the oxidative process. 

It has been long known that oxidative enzymes such as cyclooxygenase, lipoxygenase (LOX), and cytochrome P450 take part in lipid peroxidation and release metabolites that are alleged to be detrimental to human health and food quality if it is related to omega-6 polyunsaturated fatty acid (PUFA) and cholesterol. LOX-mediated metabolites from omega-3 PUFA are, however, favorable in the early events of disease progression. In contrast, non-enzymatic lipid peroxidation initiated and propagated by free radical and/or reactive oxygen species is lesser known. The omega-6 PUFA metabolites released, namely isoprostanoids, isofuranoids, and aldehyde products, are used as biomarkers of in vivo oxidative stress and act as bioactive molecules that trigger inflammation and vascular related diseases. Isoprostanoids of omega-3 PUFA were initially considered biomarkers but more recently have been regarded as bioactive molecules that are health-benefiting, particularly in regulating cardiac muscles. 

In this Special Issue, the diverse topics related to non-enzymatic lipid peroxidation of PUFA are introduced. PUFAs are important component in the brain and any oxidative stress potentially lead to modification. Signorini et al. [1] have emphasized distinct characteristics that need to be taken into account when considering isoprostanoids as biomarkers in neurological diseases. Furthermore, Millán et al. [2] have endorsed this, and have highlighted that the use of mass spectrometry is fundamental in the measurement of these biomarkers, especially in new-borns where samples are scarce and the concentration is low. The group indicated a quick and robust method would better assist in early decisions in the diagnosis of and intervention with new-borns during the neonatal period.

The development of isoprostanoids is not limited to mammalians. Medina et al. [3] suggested them to be biomarkers for plants and quality control biomarkers of agricultural products such as nuts, seeds, rice, and algae. These isoprostanoids are proposed to be bioactive in vivo and to take part in preventing pathological processes. Leung et al. [4] have observed that depending on the cooking method, the profile of isoprostanoids is different. Interestingly, bioactive isoprostanoids of omega-3 PUFA which are cardioprotective have been observed to be enhanced during the cooking process.

Lastly, without doubt, the coexistence of antioxidants and PUFA is necessary in humans. That PUFA is prone to lipid peroxidation can be mitigated by the presence of antioxidants such as polyphenols. Crauste et al. [5] have displayed a novel approach by conjugating polyphenol to PUFA for the better availability of PUFA and the polyphenol in vivo and have shown a vigorous antioxidant effect in retinal cells. This symbiotic effect is valuable as it will have dual benefits for human health. 

This issue represents the importance of non-enzymatic lipid peroxidation in nutrition and health. Certainly, there is great room for investigation to explore its role at molecular level.
